# Diagnóstico Complicado: Uma Corda Aberrante da Valva Mitral

**DOI:** 10.36660/abc.20220573

**Published:** 2023-02-23

**Authors:** Marina Santos, Mariana Paiva, Joana Ferreira, Sara Guerreiro

**Affiliations:** 1 Dr. Nélio Mendonça Hospital Madeira Portugal Dr. Nélio Mendonça Hospital, Madeira – Portugal; 2 Santa Cruz Hospital Lisboa Portugal Santa Cruz Hospital, Lisboa – Portugal; 3 Setúbal Hospital Centre Setúbal Portugal Setúbal Hospital Centre, Setúbal – Portugal

**Keywords:** Cardiopatias Congenitas, Septo Interatrial, Staphylococcus Aureus, Insuficiência da Valva Mitral, Endocardite Bacteriana, Diagnóstico por Imagem/métodos

## Caso

**Figura 1:**
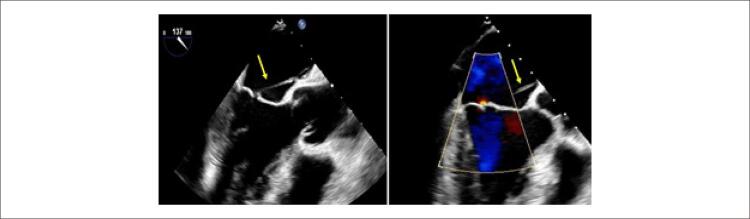
– Imagens 2D de ecocardiograma transesofágico que mostram estrutura fina e bem delineada (seta amarela), com origem no septo interatrial até à ponta do folheto anterior da valva mitral. Ausência de regurgitação significativa através de Doppler cor (à direita).

**Figura 2:**
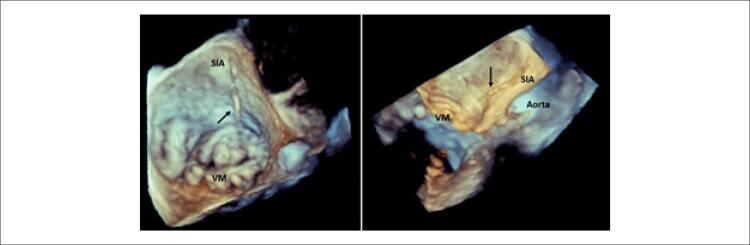
– Imagens 3D de ecocardiograma transesofágico confirmando a presença de corda anómala conectando o segmento A3 da valva mitral (VM) à região média do septo interatrial (SIA).

Apresenta-se o caso de um homem de 52 anos, com antecedentes de hepatite-C e abuso de drogas endovenosas, que estava internado no serviço de infectologia de outro hospital por espondilodiscite e abcesso no músculo psoas. Durante o internamento isolou-se *Staphylococcus aureus* em culturas de sangue, urina e líquido cefalorraquidiano, pelo que se iniciou antibioterapia dirigida. Realizou-se ecocardiograma transtorácico, que não mostrou sinais de vegetações, abcessos ou fístulas. Por manutenção de febre e suspeita de endocardite infeciosa (EI), foi submetido a ecocardiograma transesofágico (ETE). O ETE, por sua vez, apresentava uma estrutura filamentosa e móvel apensa à superfície atrial da valva mitral (VM). O diagnóstico de EI foi assumido e após sete dias de terapêutica o doente foi referenciado ao nosso hospital (terciário) para repetição de ETE. Nas imagens bidimensionais (2D) encontrou-se uma estrutura fina, bem delineada, com origem no septo interatrial (SIA) até à ponta do folheto anterior da VM, não condicionando regurgitação significativa ( Figura 1 , vídeo suplementar 1). Com imagem tridimensional (3D) confirmou-se a presença de uma corda anómala conectando o segmento A2 da VM à região média do SIA ( Figura 2 , vídeo suplementar 2). Não havia evidência de EI e esta estrutura era compatível com uma corda da VM com inserção anómala no átrio esquerdo.

A inserção atrial aberrante de corda mitral é uma anomalia congénita rara, com relevância clínica incerta. Alguns relatos de caso destacam esta anomalia como causa de regurgitação mitral importante, onde a cirurgia pode estar indicada.^1 , 2^ Além disso, está descrito um caso de endocardite infeciosa envolvendo uma corda anómala da VM.^3^ No entanto em doentes sem insuficiência mitral significativa ou infecção ativa, o reconhecimento adequado dessa anomalia é essencial para evitar tratamentos desnecessários ou diagnósticos incorretos.^4 , 5^
