# Supplementary material for: Diagnóstico Complicado: Uma Corda Aberrante da Valva Mitral
Source: Arq Bras Cardiol. 2023 Feb 23;120(3):e20220573. [Article in Portuguese] doi: 10.36660/abc.20220573 (PMC9972894; doi:10.36660/abc.20220573)
Supplement: Translation

# Translation: Tricky Diagnosis: An Aberrant Mitral Valve Chord

**Authors:** Marina Santos, Mariana Paiva, Joana Ferreira, Sara Guerreiro

**Affiliations:** 1 Portugal Dr. Nélio Mendonça Hospital, Madeira – Portugal; 2 Portugal Santa Cruz Hospital, Lisboa – Portugal; 3 Portugal Setúbal Hospital Centre, Setúbal – Portugal

**Keywords:** Heart Defects, Congenital Atrial Septum, Staphylococcus Aureus, Mitral Valve Insufficiency, Endocarditis Bacterial, Diagnostic Imaging/methods

## Case

We present the case of a 52-year-old man with hepatitis C and a history of intravenous drug abuse, admitted to the infectology department with spondylodiscitis and psoas muscle abscess. Staphylococcus aureus was isolated from blood, urine, and spinal fluid cultures, and the transthoracic echocardiogram was negative for vegetation, abscess, or fistula. The transesophageal echocardiogram (TEE) showed a filamentous and mobile structure attached to the atrial surface of the mitral valve (MV), and a diagnosis of infective endocarditis was presumed. Seven days later, the patient was referred to our tertiary institution for a detailed TEE. In 2D images, we found a thin, well-delineated strand connecting the interatrial septum (IAS) to the tip of the anterior mitral leaflet without significant regurgitation ( Figure 1A , supplementary video 1). 3D imaging confirmed the presence of an anomalous chord connecting the A2 scallop of MV to the middle of the interatrial septum ( Figure 1B , supplementary video 2). There was no evidence of infective endocarditis, and this strand was found to be compatible with an anomalous left atrial MV chord.

**Figure 1:** – 2D transesophageal echocardiogram images showing a fine, well-delineated structure (yellow arrow) originating from the interatrial septum to the tip of the anterior leaflet of the mitral valve. There is no significant regurgitation on the color Doppler (right).

**Figure 2:** – 3D transesophageal echocardiogram images confirm the presence of an anomalous chord connecting the A3 segment of the mitral valve (MV) to the middle region of the interatrial septum (IAS).

Anomalous insertion of the left atrial (LA) mitral valve (MV) chord is a rare congenital anomaly with unclear clinical relevance. Some reports highlight this anomaly as a cause of significant mitral regurgitation where surgery may be indicated.^1 , 2^ Additionally, complex mitral valve endocarditis involving an anomalous LA MV chord has been recorde.^3^ However, in patients without significant mitral regurgitation or active infection, adequate recognition of this anomaly is essential to avoid unnecessary treatment or incorrect diagnosis.^4 , 5^
